# Implementing a digital human resources management tool in the government health sector in Bangladesh: a policy content analysis

**DOI:** 10.1186/s12913-021-07304-4

**Published:** 2021-12-16

**Authors:** Dipika Shankar Bhattacharyya, Goutam Kumar Dutta, Iffat Nowrin, Sohana Shafique, Md. Zahidul Islam, B. M. Riazul Islam, Iqbal Anwar

**Affiliations:** 1 Health Systems and Population Studies Division, icddr,b, 68, Shaheed Tajuddin Ahmed Ave, Mohakhali, Dhaka, Bangladesh; 2 Coordination and Support Centre, Directorate General of Health Services, Dhaka, Bangladesh

**Keywords:** Health policy, Human resources for health, Information communication and technology, Policy content analysis, Bangladesh

## Abstract

**Introduction:**

In Bangladesh, to address the challenges of ensuring adequate human resources for health (HRH), the government began implementing a digital tool for HRH management in 2017. However, evidence suggests institutionalizing such tools in low-and-middle-income countries is impeded by policy aspects like implementation strategy and poor regulatory framework. Therefore, we aimed to explore factors in the current policy landscape that might facilitate and challenge the implementation of the tool in Bangladesh.

**Methods:**

We conducted a review of policies related to ICT implementation and human resources management in the health sector in Bangladesh using qualitative content analysis method. Ten policies have been identified, and extensive reading was done to ascertain common themes and patterns. A document analysis matrix was developed to synthesize and help interpret the findings.

**Results:**

Regarding facilitators, strong upstream level commitments were reflected in the content of policies in terms of setting out specific objectives, targets, timelines, and budget allocation. However, the lack of explicit monitoring strategy and extent of stakeholders’ engagement was not well-defined, ultimately creating chances for impeding downstream implementation. In addition, effective coordination among stakeholders and different HRH and ICT policies could be strengthened.

**Discussion:**

Findings support the current discourse that national commitment plays a vital role in the integration of ICTs in health services. However, well-defined monitoring strategy and inter-ministry and intra-ministry policy coordination are crucial.

**Supplementary Information:**

The online version contains supplementary material available at 10.1186/s12913-021-07304-4.

## Introduction

Human Resources for Health (HRH) is regarded as the heart of any health system [1] which plays a pivotal role in ensuring high-quality health care services and thus improving population health outcomes [2]. The recognition that population health outcome is, by and large, related to the number, quality, and equitable distribution of HRH, has drawn policy interest in recent years that resulted in enhanced advocacy, investment, and action at the global, regional, and national levels [3–5]. Several international calls have advocated strengthening health systems by documenting and regular updating of health workers’ numbers, deployment, and movement status [5]. However, there is a lack of organized single nationally representative data source in majority of Low-and-Middle-Income-Countries (LMICs) that captures the various dynamics of HRH stocks and flows for management decision support [5]. In general, HRH information can be gleaned from several databases such as national censuses, labor force and employment surveys, health facility surveys, and administrative databases of the public and private sector organizations. However, many of them are not designed for the specific purpose of HRH planning and management.

Dussault et al. [3] mentioned that lack of evidence-base in HRH policymaking may cause an imbalance in production, inappropriate skill-mix, and inequitable distribution of HRH. As a result, it may leave many rural and hard-to-reach areas to be underserved. These crises are particularly evident in LMICs [6], where health systems governance and stewardship is poor. In Bangladesh, HRH management faces many challenges and has been discussed in the health system and policy discourse over a long time [7]. The World Health Organization (WHO) has identified Bangladesh as one of the countries with “severe health workforce shortage”. Moreover, the existing HRH is inequitably distributed- huddled disproportionately in urban areas [8, 9]. Therefore, efficient HRH planning and management is one of the priority issues in Bangladesh to achieve universal health coverage (UHC) and health-related targets of the Sustainable Development Goals (SDGs) [10, 11].

To improve HRH management, developing a comprehensive system for collecting, organizing, and disseminating HRH information is the vital first step [1, 12]. This was strongly emphasized in the first global forum for HRH, in Kampala, in 2008 [13]. A functional model HRIS uses standardized methods for capturing and analyzing data to store and analyze updated, correct and complete information on the health workforce. In addition, this helps policymakers create a solid evidence-base by linking the health workforce data with other health indicators, e.g., disease burden, service utilization and health status indicators. Several international and regional calls have highlighted the importance of a coordinated and standardized health workforce information system [13–19].

In line with these Global recommendations, the Bangladesh government has started implementing an internet-based ICT tool - known as Human Resource Information System (HRIS) – in 2017 to improve human resource management in the health sector. Anchored within the Management Information System (MIS) of the Directorate General of Health Services (DGHS), the HRIS tool is for creating a robust central platform that incorporates and process all HRH data of government health system from all over the country [20].

However, global evidence suggests that institutionalizing any ICT tool in healthcare organizations in resource-poor countries is impeded by multiple factors, including policy-level regulatory issues and implementation level health system’s bottlenecks [5, 21, 22]. Therefore, it is essential to understand to what extent the existing policies are aligned to support the execution of digital online HRIS in Bangladesh. In this context, we conducted an exploratory study to document the policy-level factors that facilitated or constrained the implementation of the ICT-based HRIS tool for improving HRH management in Bangladesh.

## Methods

### Data source and search strategy

Using the qualitative content analysis method [23], we reviewed government policies, strategies, and plans on ICT implementation and human resources management in Bangladesh.

In order to identify all publicly available policy documents related to ICT integration in HRH management in Bangladesh, two search strategies were used. Firstly, online searches were performed in Google, Google Scholar, and websites of DGHS, ministry of health and other relevant ministries e.g. ministry of law, ministry of public administration, ministry of Science and Information and Communication Technology. Searches were conducted from October 2019 to January 2020. The search terms included “Bangladesh” “Government” “National” “Ministry of Health and Family Welfare” “doctor” “non-medical staff” “Information Communication and Technology” “policy” “strategy” “implementation” “operational plan” “Human Resources for Health” “Health Management Information System” “recruitment” “deployment” “promotion” “transfer” “health financing”. The search terms were selected in consultation with the representatives (ZI, BMRI) from the government working in ICT integration in HRH management. Two team members (GKD and IN) searched independently to find the latest version of the documents. Once the search was completed, the team members then sat together to collate the list of policy documents after excluding the duplicates.

In addition to the web-based search, we used a stakeholder-based search strategy. Key staff at the ministry and DGHS and prominent health system researchers involved in HRH policy and implementation of HRIS were contacted. The stakeholders suggested the names and contact details of relevant policy stakeholders at the ministry and directorate level. Then, through in-person meetings and telephone conversations, a series of discussions were held with them by the research team members to obtain new policy documents and to validate the list of the most recent policy-related documents. The list was then finalized by the research team members (DSB, GKD, ZI, BMRI, IA) after thorough discussion with stakeholders.

**Fig. 1:**
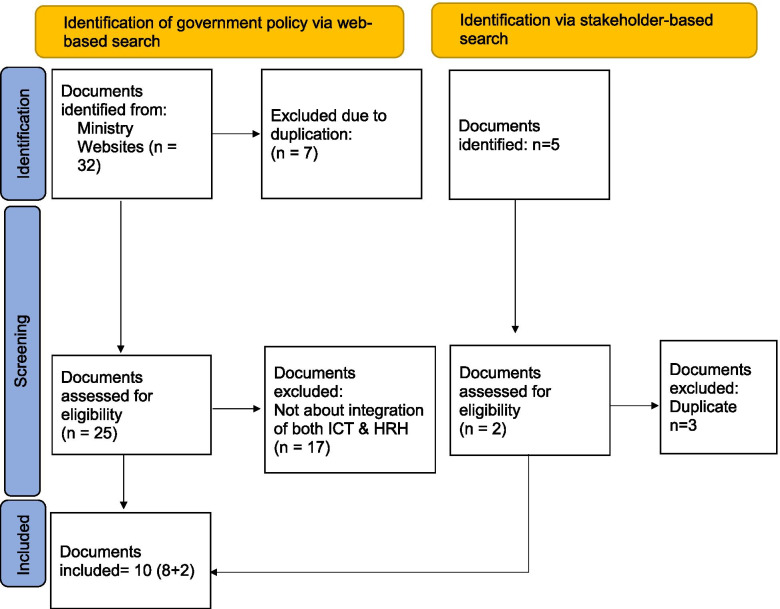
Flowchart of document selection process

Policies that have specific mentions about the integration of ICT tool in the management of HRH in Bangladesh have been included in the review. Policies that have specific HRH issues, e.g., posting and transferring certain staff in the hospital, but do not have implications in the integration of ICT tool were excluded. Similarly, the ICT-related policies that do not converge with HRH management were excluded from the review. The flowchart for the selection of documents is shown in Fig. 1.

### Data extraction process

Once the documents have been finalized, the authors undertook an extensive and organized reading of the policy documents to identify codes and patterns [23–25]. All the included policies were read and re-read line by line to achieve complete comprehension and to refine the findings as per the study objectives. The policy document review was performed independently by three authors (DSB, GKD, and IN) as per the mentioned objectives. The independent reviewers separately coded the policy documents according to their review. Afterward, all of them discussed through several meetings to mitigate the disagreements and reach on a consensus. Extracted information was sorted under selected themes as per the study objectives. Atlas.ti 7.5 software was used for coding the texts, storing the findings, and organizing the systematic readings. At this stage, a data analysis matrix was developed. In order to ensure the appropriateness of the matrix, we formed a team with a diverse background that consisted of government representatives working in the implementation of the digital tool (ZI, BMRI), government health system experts (SS, IA), review experts (IN) and researchers (DSB GKD). A thorough discussion was made to finalize the matrix.

For all the included documents, the columns contained five key data points (and some sub-themes where needed):

- Name of the policy/strategy/order
- Source of the information
- The year when the policies/orders/gadgets were published
- Facilitators
  - ✓ Commitment for ICT based HRM
  - ✓ Actors /implementing departments or organizations (Leadership)
  - ✓ Allocated budget
  - ✓ Resources for training and other support
- Challenges/barriers
  - ✓ Stakeholder engagement and role specification
  - ✓ Monitoring strategy
  - ✓ Coordination

### Data analysis

For data analysis, the information from the data display matrix described above was synthesized. Then the study investigators themselves conducted the document review and detailed analysis. It is worth mentioning that many of the policy documents were in Bengali, and all the authors were fluent in both Bengali and English to extract, interpret and synthesize the information. In this regard, the key themes from all the policies were first identified, and then the authors conducted further analysis through synthesizing this information to explore and understand policy level factors that supported or constrained the implementation of the HRIS tool for improving HRH management in Bangladesh context. The policy review was guided by the “Policy Analysis Framework” proposed by Collins [26] following eight steps: 1) define the context; 2) state the problem; 3) search for evidence; 4) consider different policy options; 5) project the outcomes; 6) apply evaluative criteria; 7) weigh the outcomes, and 8) make the decision. According to this framework, the first step is about describing a profile of the given country. The second step contains the definition of the health problem, while the third step involves explicitly focusing on the policy issue to be examined and collecting related evidence to be examined. The next step requires setting up the policy linkages to other relevant contextual factors, e.g., other policies in the country. The next step is to assess the outcomes in line with the available contextual factors. Then applying the evaluative criteria in terms of relevance, progress, efficiency, effectiveness, and impact; however, the choice of application criteria depends upon the subject matter to be studied. The following steps are about weighing the outcomes and making decision based on this. We have covered steps 1–3 in the introductory section of this paper. In order to present the findings of steps 4-8, the authors have broadly categorized the result into two broad themes; i) policy level facilitators to integrating ICTs in HRH ii) Lacunae in policy and the scope for further strengthening.

## Results

In total, 10 National level policies, strategies, and plans that focused on integrating ICT in HRH Management in Bangladesh were included in the review (Table 1).

**Table 1:** List of policies reviewed

SL No.	Name of policies	Implementer	Published year
1	The National ICT Policy, 2009 [27]	MoSICT	2009
2	The National Health Policy, 2011 [28]	MoHFW	2011
3	Program Implementation Plan (PIP), 2011-16 [29]	MoHFW	2011
4	Bangladesh Health Workforce Strategy, 2015 [30]	MoHFW	2015
5	Program Implementation Plan (PIP), 2017-22 [31]	MoHFW	2017
6	The National ICT Policy, 2018 [32]	MoSICT	2018
7	Health Care Financing Strategy, 2012-32 [33]	Health Economics Unit, MoHFW	2012
8	The HRIS User Manual [34]	Management Information System, DGHS, MOHFW	2013
9	Election Manifesto on digital Bangladesh [35]	Bangladesh government ruling party	2008
10	Mapping of Ministries by Targets in the implementation of SDGs aligning with 7th Five Year Plan (2016-20) [36]	General Economics Division (GED) Planning Commission	2016

All the policies included in the review were published between 2009 and 2018 (27-36). Among them, four policies were essentially focused on the use of ICT tool in HRH management [29–31, 34]; others have relevance or implications for introducing ICT in HRH management- [27, 28, 32, 33, 35, 36]. Among the included policies, six were from the Ministry of Health and Family Welfare, two from the Ministry of Science and Information Technology, and one was from the Planning Commission. In addition, the government’s election manifesto has been regarded as the central vision for how the digitalization processes and initiatives would be taken in various sectors, including health, in the country. All these policies were identified through detailed discussions with relevant stakeholders working on HRH issues in public or in the private sector in Bangladesh.

### Policy level facilitators to integrate ICTs in HRH

The key issues identified as the facilitating factors are as follows.

#### Upstream level decisions for integrating ICT and health

Strong upstream level commitments have been found in the policy landscape regarding ICT integration across all government departments, including health. For example, the government manifesto of “Digital Bangladesh,” formulated in 2008, advocates for ensuring an ICT-based society where all government, non-government, and semi-government organizational activities would be performed using ICT networks to ensure equitable and comprehensive development by 2021 [35]. Placing this “Vision 2021 Agenda” as a guiding principle of the government, all ministries of the government of Bangladesh began to thrive using digital platforms for providing high-quality need-based services for the citizens of the country.

In addition, the Government of Bangladesh expressed commitment to achieving Universal Health Coverage (UHC) by 2032. With this aim, the ‘health care financing strategy’ was formulated by the Ministry of Health and Family Welfare (MOHFW) in 2012, which acknowledges that, along with other factors, human resource development, as well as proper utilization of ICT, is imperative for achieving UHC goals [33]. Furthermore, Bangladesh has signed the United Nations Sustainable Development Goals (SDGs), which obligates the Government of Bangladesh to make more substantial investments for improving HRH management. All these upstream policy level commitments created a conducive environment to integrate ICT for strengthening health systems. The commitments are reflected in recent advancements in MOHFW – such as the introduction of online DHIS2, Open MRS, HRIS, etc., for ensuring ‘measurement and accountability and ‘data for decision making in the government health system.

#### Commitments for ICT based HRM

The upstream level commitments have been reflected in the content of relevant policies and strategies of the Ministry of Health and Family Welfare (MOHFW) and other related ministries. The National ICT Policy 2009 formulated by the Ministry of Science and Information & Communication Technology (MoSICT) is a prime example. This policy was formulated to function as a “binding guide” for adopting ICTs by all other government ministries to improve the efficiency and effectiveness of government interventions. The national ICT policy provided a particular emphasis on the health system. Strategic guidance was given to improve the health care delivery system by introducing e- and m-health in service delivery, monitoring, and evaluation. In addition, under the strategic theme 7.1, the ICT policy recommends developing, updating, and using a comprehensive national database for all HRH, including doctors, nurses, paramedics, and alternative medical practitioners in the country. The policy recommends that this comprehensive and dynamic database be used for all kinds of HRH management activities, including recruitment, posting, transfer, retention, and retirement. Importantly, creating this database was a priority short-term task, mentioned as “need of the hour,” which was supposed to be implemented within 18 months of policy formulation. Therefore, the national ICT policy was the first impetus for building and using an ICT-based tool for improving HRH management in the government sector [27].

One of the main goals of The National Health Policy (2011) was to ensure the best utilization of ICT tools for improving overall health systems performance. Accordingly, strategy#13 of this policy has elaborate recommendations for establishing an integrated management information system using a computer network for planning, implementing, and monitoring all health programs in the public sector [28]. However, there was no specific content in National Health Policy for integrating ICT in HRH management.

The Program Implementation Plan (PIP) of the “4th Health, Population and Nutrition Sector Development Programme (HPNSDP) 2011-16” is the first policy document in the health sector that took specific decisions to provide accurate and up-to-date HRH information to policy makers and all relevant stakeholders for better HRH planning and management. The operation plan (OP) on Human Resources Management (HRM) of the 4th HPNSDP identified problems for not having a central information system on HRH. It thus endorsed a specific decision for establishing an ICT-based HRH information system to ensure real-time data for management decision support and planning. This policy set a specific target to establish a central HRIS within 2014 and utilize it for HRH management by mid-2016 [29]. More importantly, there were clear recommendations for updating the National Health Workforce Strategy to promote evidence-based HRH planning and management. Based on this recommendation, ‘The Health Workforce Strategy 2015’ was formulated by MOHFW, in which “Health Workforce Information System (HRIS)” was taken as one of the five main thematic areas.

Under HRIS thematic area, the significant commitment was to establish a comprehensive central health workforce information system to promote evidence-based decision-making in HRH management [30]. This commitment was further enforced in the PIP of the following health sector program ‘the Health Nutrition and Population Sector Program (HPNSP) 2017- 2022. In this PIP, three major activities related to the integration of ICT in HRH management were identified, which are to be accomplished by 2022- i) Establishing one central HRIS that is linked to all agencies; ii) Capacity building from central to periphery level for the institutionalization of the online HRIS and iii) Using HRIS data for evidence- based planning and decision-making, and all these activities are to be completed by 2022 [31]. The PIP of HPNSP (2017-2022) has identified the DGHS as the first initiator, and other departments within MOHFW have been advised to introduce HRIS for better HRH management. It is also worth mentioning that the Management Information System (MIS) of DGHS has developed a manual with specific instructions on how to implement the HRIS tool at the local level and how national-level policy and decision-makers can authorize and extract necessary data from the tool. The guideline has been uploaded to the HRIS log-in website in Bengali so that stakeholders from the national and local levels can easily understand this [34].

#### Budget allocation

The PIPs of the third and fourth health sector program has allocated adequate budget for establishing and utilizing comprehensive ICT-based HRH management tool. In PIP (2011-16), a total of 14,747.00 lakhs (USD 17.393 million) was allocated for the development and management of HRH. In this budget, specifically, 670.00 lakhs (USD 0.790 million) was allotted for developing the Human Resources Information System (HRIS) and automation of the human resources management process [29]. In the next PIP (2017-22), a total of 9982.47 lakhs (USD 11.770 million) was allocated for the overall improvement of HRH management. From this amount, 2193.00 lakhs (USD 2.587 million) was allocated for implementing and monitoring the HRIS tool centrally. It is important to mention that this policy declared the explicit allocation strategy of the budget for five years. According to the policy, taka 62.00 lakhs (USD 0.073 million) was allotted for establishing one central HRIS that is linked to all agencies within MOHFW; 61.00 lakhs (USD 0.072 million) for establishing monitoring framework for implementation of Health Workforce Action Plan, 61.00 lakhs (USD 0.072 million) to ensure use of HRIS data for evidence-based planning and decision making, 1570.00 lakhs (USD 1.852 million) for research/ survey/ study and 439.00 lakhs (USD 0.518 million) for seminar/ conference/ training required for implementation [31].

### Lacunae in policy and scope of strengthening

#### Stakeholder engagement

There are specific recommendations across the policies to engage different stakeholders for implementing the HRIS. In general, the government agencies, semi-government institutes, and in some cases, private sector organizations were identified as stakeholders to implement the HRH-related policies. However, there was no specific declaration about the role of the implementing bodies, and there was limited information about the extent of engagement of other stakeholders outside government. For example, according to the National ICT policy 2009, Bangladesh Computer Council or its successor organizations are identified as the implementing actors. However, the specific role of each implementing body, such as other ministries and departments, was not explicitly mentioned in the policy. The policy also identified the private sector as one of the primary actors implementing the HRH database (item#218, under Objective#7). However, there is no clear role clarification on how the private sector players will be engaged in the planning and implementation [27].

The 4th Health, Population and Nutrition Sector Programme (HPNSP 2011-2016) declared the importance of engaging multiple stakeholders in HRH management with their full involvement as per requirements. Nevertheless, there is not enough reflection about the scope of engagement of multiple stakeholders for specific tasks. Similarly, ‘Bangladesh Health Workforce Strategy - 2015’ recommended some short-term, mid-term, and long-term strategic interventions to strengthen the health workforce information system. In this regard, both government agencies and private sector actors were identified as key stakeholders. However, there is no clear guideline on the ways to integrate all these stakeholders for a comprehensive national database. Monitoring the private sector data of HRH has been identified as one of the major challenges in this strategy.

#### Implementation and monitoring strategy

The 3rd HPNSDP (2011-16) recommended developing and establishing a Central Human Resource Information System (CHRIS) encompassing all the directorates and departments of MOHFW by 2016 [29]. However, the subsequent 4th HNPSP (2017-22) mentioned that the HRH data was not made available in a single place from all over the country [31]. There was no specific reason mentioned regarding how establishing a central HRIS for all directorates was impeded. This policy (PIP 2017-22) also mentioned that, in establishing comprehensive HRIS under the 3rd sector program, the ownership of MOHFW was not prominent. This is also reflected in the Logical Framework of the 4th PIP, as under the component-5 “Important Assumption” was taken as “Health Service Division of the MOHFW has control on CHRIS based at MIS department of DGHS” [31]. This assumption indicates that while the DGHS has a well-developed HRIS for capturing their countrywide HRH, there was a lack of implementation plan of other agencies within MOHFW.

The Health Workforce Strategy 2015 and the two PIPs (2011-2016 and 2017-2022) mentioned a long-term plan to assess the implementation, monitoring, and reviewing of the HRIS. However, the other policies and strategies lack such strategy to evaluate the appropriateness of the design of the tool as well as the coordination mechanism among stakeholders in the implementation process. The National ICT Policy did not provide any clear idea about the budgetary allocation for developing and using the database for HRH management. Similarly, the Bangladesh Health Workforce Strategy 2015 recommended a step-wise approach for establishing and using the HRIS, but the action plans were designed without referring to any specific budget.

#### Coordination among policies

All these national-level policies related to ICT use in the management HRH were formulated gradually one after another over the last 10-12 years. However, there are certain gaps with regard to coordination among these policies. For example, the National ICT policy adopted in 2009 [27] and later updated in 2018 [32] had a component on ‘update and database for management of human resources to be implemented as a short term task (within 18 months of policy formulation). However, that policy’s progress or implementation status was not reflected in the subsequent MOHFW policies such as PIPs of the third and fourth Health Sector Programme [29, 31] or Health Workforce Strategy 2015 [30]. Even it was not explicitly mentioned in those policy documents.

## Discussion

The study findings suggest that upstream national policies of the ministry of health and family welfare, ministry of ICT, and ministry of science and technology with a clear mandate and timeline facilitated implementing the ICT tool for HRH management in the government health sector. The findings support the current knowledge base that the process of health-related policy formulation is largely influenced by the upstream level political priorities of the national government and international agendas [37, 38]. The “Digital Bangladesh” agenda of the government has been translated into the perspective plan of 2021, which ultimately guided the relevant ministries to take policy initiatives for digital solutions. This finding resonates with recent studies that show there are a number of sound policies and relevant initiatives to incorporate ICTs in the health sector in Bangladesh [20, 39]. The latest survey of the World Health Organization on digital health has also emphasized that well-articulated national e-health policy is a prerequisite for strengthening the health system by applying appropriate ICT solutions and initiatives [40]. For integrating ICTs in HRH management, the importance of upstream level policy is evident from studies in other LMICs. For example, in Mozambique, a strong commitment of national leadership was instrumental in building a robust human resources information system in the public sector [41]. On the other hand, in Pakistan, the implementation of such ICT tools in HRH was hampered mainly due to inadequate input from national-level policies [42].

The study has highlighted the importance of budgetary allocation for implementing the ICT tool in improving HRH management. Establishing a functional HRIS is a complex and challenging task in resource-poor settings. It involves a series of inter-related and consecutive actions- e.g., data collection, entering, analyzing, reporting- by separate entities at different tiers of health systems [5]. Therefore, for ensuring a sustainable mechanism-solution in HRH management, continual government fund allocation is a prerequisite. In this regard, the current policies in Bangladesh has provided necessary guidance and allocated sufficient financial resources for the installation and subsequent training on the tool for different health system actors. The necessity of ensuring budget allocation for the implementation of ICT tool in HRH management has been reported from other countries also. For example, in Tanzania, the issues of maintenance and proper use were integrated into the e-Health strategy of the ministry of health to ensure the availability of funds to develop a sustainable HRH information system [43].

The study findings also emphasized that although the policies have identified the key stakeholders, there is a need to give attention to making stakeholders on board with clear role clarification. This finding is congruent to a recent assessment of sector-wide approach in Bangladesh [44]. The government ministry’s planning process is inherently centralized, which hampers decentralization and poses minimal scope for the inclusion of non-state sector stakeholders in the health policy process [44]. Especially in terms of implementing ICT tool in the Bangladesh health system, the absence of stakeholders’ motivation was discussed as one of the significant barriers in the previous study [45]. The study demonstrated that in Bangladesh, establishing a central database for all health workers within different directorates of MOHFW was not fully accomplished as targeted. Besides, incorporating HRH data from private sector organizations was also challenging. A similar situation prevails in other countries. Previously, the WHO hosted a technical meeting to bring together the experiences and initiate discussion on ways to promote comprehensive country health workforce information, including private sector data. The meeting emphasized that while establishing such systems, the central concern is organizing the scattered data in one place and, more importantly, bringing different stakeholders together on a single platform [14]. Therefore, developing and sharing a framework of stakeholder engagement for establishing and maintaining the HRH information systems might be helpful for all stakeholders to come to a consensus around the implementation.

This study was directed towards exploring the extent to which the current policies can support the implementation of HRIS in Bangladesh. The finding suggests HRIS implementation is largely influenced by the idea that it will help policymakers make the evidence-based decision around HRH issues in Bangladesh. A study from Kenya suggests that, in addition to day-to-day decision making, the implementation of such tool has the potential to impact policies as data from the Kenyan Health Workforce Information System (KHWIS) helped government to formulate policy to extend the retirement age of nurses since there were more nurse retiring than the upcoming young nurses in the health system [46]. Therefore, in Kenya, it was evident that policy formulation and implementation of ICT tool in HRH is a two-way relationship. However, in our policy review, we did not find any documentation regarding how the HRIS implementation influences policy-making in Bangladesh.

A growing number of implementation research findings suggest that regular monitoring and evaluation have a strategic role in improving policy decisions’ relevance, efficiency, and effectiveness in the LMIC settings [47, 48]. Therefore, monitoring and evaluation need to be integrated into the design of policy implementation so that poor enforcement or resource wastage can be avoided [48]. The study finding revealed that in Bangladesh, several policy documents were formulated with specific directives on setting out targets, timelines, and budget allocation for establishing HRIS. However, effective coordination among different health workforce policies needs to be reinforced. Besides, coordination needs to be strengthened within different directorates and between private sector actors to create a comprehensive health workforce information system. This finding resembles a recent NCD policy analysis in Bangladesh, concluding that despite the availability of many policies, significant coordinated attention is warranted to monitor how these policies can be effectively implemented in the health system [49]. Many countries are also envisaging similar problems. This problem arises because the weak mechanisms for providing feedback to decision-makers in the policy enactment process are often overlooked [50].

The limitation of this paper is that it is based only on the review of policies; therefore, it does not reflect the facilitators and the bottlenecks in the context of field-level implementation. FOr example, there might be some policy decisions taken by utilizing the HRIS. However, those decisions are not included here since these are not documented in the included policies in this review. Therefore, further studies will be required to explore this. It is also worth mentioning that the policy review is not a straightforward method since multiple theoretical and methodological assumptions exist on how the public policies can be investigated and understood [51]. However, this paper presented an integrated view of the policy landscape in Bangladesh regarding the implementation of an ICT-based HRH management tool in the government health sector. In addition, to create a knowledge base on the strengths of the existing policies, the findings from this review lay a foundation for policymakers to identify the options that need to be addressed in future policies. We recommend further qualitative research on the key facilitators and challenges of field-level implementation of the tool in the Bangladesh health system.

## Conclusion

The development of innovative technology brings new opportunities for strengthening health systems. However, while the technology offers new hope, there are also associated challenges with regard to how to fit the technology in the best way within the health system considering the country context. Therefore, to leverage the desired outcome of digital technology in health, a review of national policy and strategy will create an evidence base to better support innovation. From this context, the findings of this research showed that national commitment plays a vital role in the integration of ICTs in health services. However, a well-defined monitoring strategy and inter-ministry and intra-ministry policy coordination for creating a conducive environment are crucial for its strengthened implementation. In addition, more implementation research is needed for exploring field-level issues to ensure successful implementation and scaling-up of ICT tools for human resource management in the health sector of LMICs.

## Supplementary Information

**Additional file 1.**

## Data Availability

The datasets used and/or analyzed during the current study are available from the corresponding author on reasonable request.

## Abbreviations

DGHS: Directorate General of Health Services
DHIS2: District Health Information System 2
ERC: Ethical Review Committee
HPNSDP: Health Population Nutrition Service Delivery Program
HPNSP: Health Population Nutrition Sector Program
HRM: Human Resources Management
HRH: Human Resources for Health
HRIS: Human Resource Information System
HWF: Health Workforce Strategy
ICT: Information Communication and Technology
KHWIS: Kenya Health Workforce Information System
LMICs: Low and Middle Income Countries
MIS: Management Information System
MOPA: Ministry of Public Administration
MOSICT: Ministry of Science and Information & Communication Technology
NCD: Non Communicable Diseases
OP: Operation Plan
PIP: Program Implementation Plan
RRC: Research Review Committee
SDGs: Sustainable Development Goals
UHC: Universal Health Coverage
WHO: World Health Organization
